# Symptomatic methemoglobinemia in a patient with metastatic clear cell renal cell carcinoma treated with pembrolizumab and axitinib combination therapy: a case report

**DOI:** 10.1186/s13256-020-02637-w

**Published:** 2021-02-19

**Authors:** T. Anders Olsen, Dylan J. Martini, Sean T. Evans, Jamie M. Goldman, Mehmet Asim Bilen

**Affiliations:** 1 Department of Hematology and Medical Oncology, Emory University School of Medicine, Atlanta, GA USA; 2 Winship Cancer Institute of Emory University, 1365 Clifton Rd, Atlanta, GA USA

**Keywords:** Case report, Methemoglobinemia, Immunotherapy, Adverse events and renal cell carcinoma

## Abstract

**Background:**

Combination regimens that include immune checkpoint (ICI) and vascular endothelial growth factor (VEGF) inhibition have opened the door to new treatment opportunities for patients with metastatic renal cell carcinoma (mRCC). While these treatment options have provided improved tolerability and better outcomes compared to older regimens, many patients still experience a myriad of treatment-related adverse events. Given that these regimens were recently approved for mRCC, the complete side effect profile may not be fully elucidated yet.

**Case presentation:**

We report a case of a 73-year old White male with mRCC who was managed with an ICI-VEGF inhibitor combination regimen. He experienced a partial response (Fig. 1) but had side effects including symptomatic cyanosis diagnosed as methemoglobinemia which led to treatment discontinuation. Upon holding his therapy, his methemoglobinemia and cyanosis resolved.

**Conclusions:**

Combination VEGF-ICI therapy provide novel regimens for advanced solid tumor malignancies including mRCC. While shown to have improved efficacy in clinical trials, it is crucial that oncologists uncover the full side effect profile of these novel agents especially as their use becomes more standard in the management of advanced malignancies. To our knowledge, this is the first reported case of a patient experiencing symptomatic methemoglobinemia as an adverse event associated with a VEGF-ICI combination regimen. While the cause of this side effect is unclear, in this paper we attempt to elucidate a process that is in line with the mechanism of action of these therapies to explain how these agents, specifically the axitinib, could have caused the methemoglobin to rise to a symptomatic level.

## Background

Vascular endothelial growth factor (VEGF) tyrosine kinase inhibitors (TKIs) are small molecules that inhibit tyrosine kinase receptors involved in the VEGF pathway [1]. These types of TKIs are pathway-specific inhibitors of the angiogenic signaling process that cancers depend upon to obtain access to nutrients and to metastasize [2]. Blocking VEGF from malignant tissue effectively causes transient hypoxia, cutting off access to nutrients vital for oncogenesis. TKIs are integral to the treatment of numerous cancers including metastatic renal cell carcinoma (mRCC) [3]. Pembrolizumab is a programmed death-1 (PD-1) inhibitor that is in a class of medications called immune checkpoint inhibitors (ICIs). These agents increase T-cell activation, improving T-cell-mediated clearance of malignant cells and are used in several malignancies including mRCC [4–6]. PD-1 and VEGF-TKI combination therapy have shown efficacy and received FDA approval as the standard treatment for advanced RCC through the phase 3 KEYNOTE-426 clinical trial [7, 8]. This regimen, however, is not without side effects which can significantly impact patient quality of life and cause treatment discontinuation [5]. In the KEYNOTE-426 trial, the most commonly cited adverse events associated with pembrolizumab-axitinib were diarrhea, hypertension, fatigue and hypothyroidism [8]. In this report we are presenting, to our best knowledge, the first case of a patient with mRCC who developed cyanosis diagnosed as methemoglobinemia while on pembrolizumab-axitinib treatment. This case displays a rare but severe adverse event that could be related to ICI and VEGF-TKI combination therapy.

## Case presentation

**Fig. 1:**
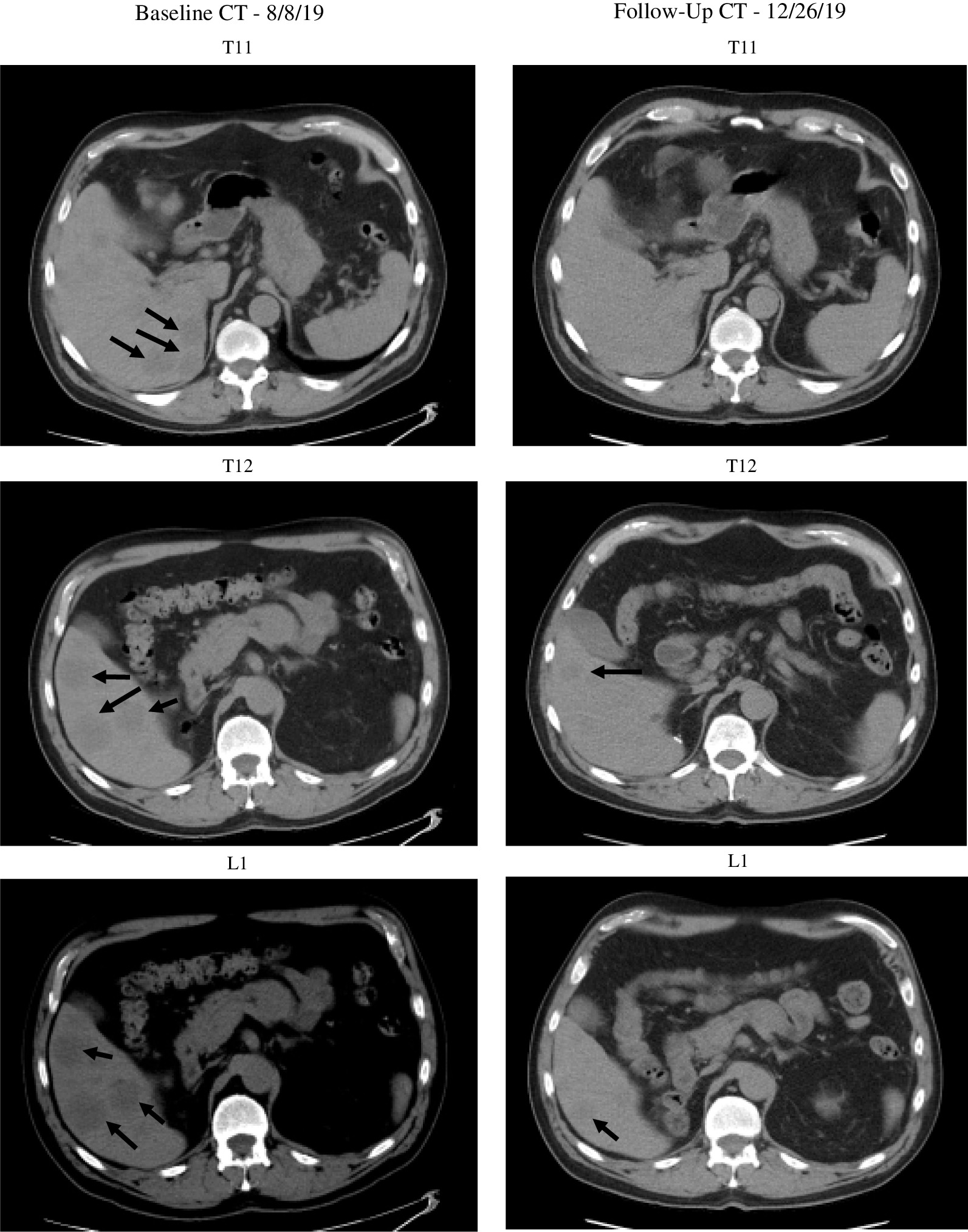
Axial contrast enhanced computed tomography scan of the abdomen and pelvis. Interval decreases in size of liver metastases with reference measurements are as follows: **T11**: 1.6 × 1.9 cm metastasis of liver towards the dome compared to baseline of 3.7 × 3.4 cm. **T12**: 1.6 × 2.2 cm metastasis towards the dome more centrally compared to baseline of 3.3 × 3.5 cm. **L1**: 2.1 × 2.1 cm metastasis inferior right hepatic lobe compared to baseline of 3.2 × 3.9 cm

A 73-year-old White male was diagnosed with pT2NxMx clear cell RCC (ccRCC) and underwent radical nephrectomy in November of 2007. Given his family history of malignancy, he underwent genetic testing and was found to be positive for CHEK2, ATM, BLM and MLH1 mutations. He then presented twelve years later with biopsy-proven recurrence of his ccRCC in the pancreas, liver and lung. He was started on combination therapy consisting of pembrolizumab 200 mg IV q3 weeks and axitinib 5mg PO BID [8, 9]. As seen in Fig. 1, his scans two months after treatment initiation showed partial response in his metastases per RECIST v1.1. His course was complicated four months after initiation by adverse events including cystitis, hematuria, fatigue, polycythemia and peri-oral cyanosis without hypoxia. His primary care physician initiated cyanosis work-up and laboratory tests were obtained. This included a complete blood-cell count with differential, which showed a positive test for methemoglobinemia displaying a percent methemoglobin level of 3.7% exceeding the 1.5% cutoff for positivity. Additional complete blood cell-count values showed a normal erythropoietin (EPO) level and an elevated hemoglobin of 18.4 with a hematocrit of 53.8. The secondary polycythemia experienced concomitantly with the methemoglobinemia was attributed to a combination of the axitinib therapy and an RCC-associated paraneoplastic syndrome. Upon holding his axitinib for 1 week, the patient’s symptoms resolved. Follow-up laboratory tests similarly showed normalization of the residual polycythemia with a subsequent hemoglobin of 15.3 and hematocrit of 47.4. While the MetHb level was only mildly elevated during the treatment period, the patient was highly symptomatic and this reaction prompted medication adjustments. He was restarted on the same combination therapy with a reduced dose of axitinib from 5 to 3 mg. His adverse symptoms recurred and were intolerable shortly after resuming. He discontinued the pembrolizumab-axitinib therapy and transitioned three weeks later to an ipilimumab 1mg/kg IVBP and nivolumab 3mg/kg IVBP combination regimen. This subsequent regimen was discontinued due to poor tolerability from immune-related adverse events including hypothyroidism and adrenal insufficiency. His follow-up scans in January showed sustained partial response, but progression of disease in June of 2020 within the liver metastasis. In September of 2020, the patient is still alive and has begun local Y90 radiation therapy and awaits upcoming surveillance scans.

## Discussion

**Fig. 2:**
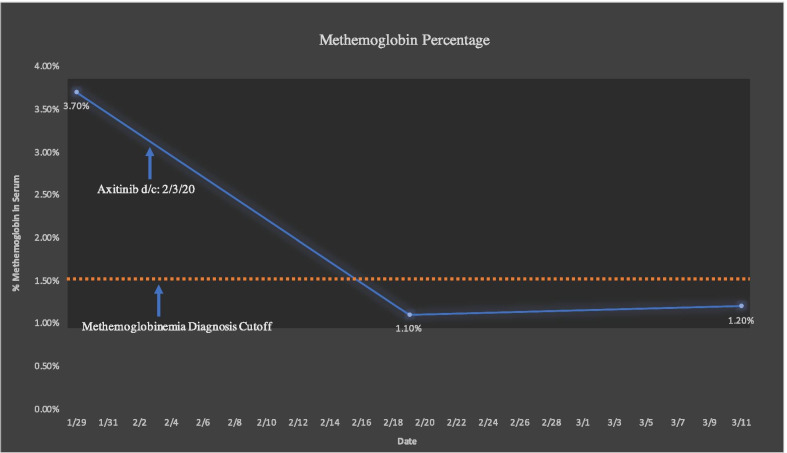
Patient’s percent level of methemoglobin relative to normal hemoglobin based on sequential complete blood-cell counts with differential

In this case, we presented a mRCC patient who had partial response to treatment with pembrolizumab and axitinib. Despite the response, he experienced recurrent cyanosis without hypoxia diagnosed as acquired methemoglobinemia, leading to treatment discontinuation. There have been no reported cases of methemoglobinemia related to either PD-1-inhibitors or VEGF-TKIs. However, our analysis of the clinical notes and labs are in line with the treating physician’s suspicion that the methemoglobinemia was most likely attributed to the axitinib. It is important to note that these symptoms resolved within seven days of holding axitinib and recurred at the lower dose. The amount of methemoglobin measured in the patient’s blood overtime can be appreciated in Fig. 2. This case could provide evidence of a rare, but significant side effect of anti-VEGF therapy when used in combination with ICI. It is important for medical oncologists to be aware of this adverse event so they can formulate the best response for their patients in the clinic.

Hemoglobin (Hb) is the oxygen carrying molecule within red blood cells (RBCs). Methemoglobin (MetHb) is a type of Hb containing an oxidized ferric (Fe^3+^) atom that is less effective at releasing oxygen compared to the normal ferrous (Fe^2+^) bound heme [10]. RBCs are frequently exposed to oxidizing agents, such as free radicals, metabolites and drug intermediates. These molecules shift the iron in their heme rings from 2^+^ to 3^+^, which forms MetHb. To combat this, the RBC contains a large reservoir cytochrome-based reducing enzymes to counteract these oxidizing particles [11]. Methemoglobinemia is a state where the percentage of MetHb relative to normal Hb rises to a symptomatic level [11]. There are two main forms of methemoglobinemia: inherited and acquired. These conditions are associated with either a genetic or an induced deficiency of these RBC reducing enzymes. The acquired form is more common and is most often due to medications such as dapsone, which form potent oxidizing agents when metabolized by cytochrome P450 enzymes in the liver [12, 13]. This patient’s medication list showed no notable agents that could have been associated with the patient’s adverse hematologic side effects. There was also no evidence of any past or familial hematologic disorders playing a role in the patient’s adverse reaction. Family history was only significant for past malignancies, which was supported by genetic mutation findings. Methemoglobinemia can also be a difficult syndrome to diagnose due to its wide spectrum of symptomology ranging from minor cyanosis to life-threatening hypoxemia [10]. This is additionally complicated within the context of RCC as RBC syndromes, such as polycythemia and anemia, are much more common amongst this patient population. For most patients, methemoglobinemia becomes symptomatic when MetHb levels rise to 10–20% rather than the 3.7% experienced by the patient in our case report. However, as stated earlier, methemoglobinemia has a wide range of symptomology and, especially for those burdened by metastatic cancer, intensive VEGF therapy and other hematologic disorders, this symptom threshold could be significantly lower for complex oncology patients.

Between the two agents comprising the patient’s treatment regimen, axitinib is more likely the culprit for the methemoglobinemia. Unlike pembrolizumab, axitinib has a short half-life of roughly 3–6 hours in vivo [14]. The patient’s markedly rapid clinical improvement upon holding axitinib supports our theory that it may have been the inciting factor in this patient’s methemoglobinemia. Additionally, axitinib can induce polycythemia and other RBC dysfunction in patients with ccRCC due to complex interactions between hypoxia inducible factor 1 alpha (HIF-1a), VEGF and EPO [15, 16]. In fact, some studies suggesting hemoglobin values could be used as biomarkers for axitinib treatment response [17]. The etiology of this patient’s methemoglobinemia is unclear at this time. However, there are a few plausible explanations for an increase in oxidizing agents that could have produced enough MetHb to become symptomatic. The partial regression noted in this patient’s metastatic lesions could have released large quantities of intracellular oxidizing components from the malignant cells as a result of a favorable response. The combination of cell lysis and T cell expansion may have led to a concomitant release of oxidizing agents that overwhelmed the RBC reducing enzymes. Additionally, while there is little evidence of axitinib or its metabolites acting as oxidizers, axitinib is metabolized by multiple cytochrome P450 enzymes which could form oxidizing agents in a similar mechanism to a dapsone-induced methemoglobinemia [14]. While the true cause of this adverse reaction is not currently known, it was likely a multifactorial event incited by an increase in oxidizing agents that overwhelmed the reducing capacity of this patient’s RBCs.

## Conclusion

Combination immune therapy provides an exciting new treatment option for mRCC patients that combines the benefits of multiple potent therapies for an even greater treatment effect. These agents can provide clinical oncologists with additional treatment options for aggressive malignancies like mRCC. However, it is crucial that medical oncologists appreciate the benefits and costs these treatment regimens impose on patients. Adverse events, such as the one presented in this case report, can be life-threatening and have significant impacts on the patient’s prognosis and quality of life. Current trials utilizing PD-1 and VEGF-TKI combination therapy have presented promising results and FDA approval for patients with advanced RCC [8]. This case presents one such example of a RCC patient who experienced a rare but important adverse hematologic event. The novelty of this presentation is both a strength and a weakness in this case study. The resulting methemoglobinemia for this patient could have equally been involved with another underlying disease process as it could be a true side effect of VEGF-TKI and ICI combination therapy. Current clinical trials should make ongoing efforts to note these uncommon side effects to better appreciate the side-effect profile of these novel agents. Cases, such as this one, can hopefully assist practicing oncologists to recognize the rare and potentially severe side effect of methemoglobinemia in mRCC patients treated with ICI and VEGF-TKI combination therapy.

### Patient perspective

“I was given pembrolizumab by intravenous means every 21 days for three months. I also was placed on 5 MG tablets of Axitinib. The combination did have great success with decreasing and/or eliminating a tumor from my pancreas and two on my liver. However, the side effects caused me to have to stop the therapy. The first symptom was a loss of appetite which in turn lead to fatigue, tiredness, loss of weight, and loss of voice volume. There was no sickness, I just didn’t feel well. My lips and eye whites turned blue and I was ultimately diagnosed with methemoglobinemia. I was weak, needed weekly infusions of fluid, and ultimately had to quit the therapy after three months. There was never any pain from anything. Dehydration became my biggest issue as I spend most of my time sleeping and not wanting to eat. I found success managing this situation with my general practitioner who is an internist specializing in kidney and diabetes. It is best to start this journey with a good oncologist that will take the time to determine what is happening with your systems. I was fortunate enough to find great doctor(s)."

## Data Availability

All data generated or analyzed during this study are included in this published article [and its supplementary information files].

## Abbreviations

mRCC: Metastatic renal cell carcinoma
ccRCC: Clear cell renal cell carcinoma
Hb: Hemoglobin
MetHb: Methemoglobin
PD-1: Programmed cell death protein-1
VEGF: Vascular endothelial growth factor
HIF-1a: Hypoxia inducible factor-1-alpha
Fe^3+^: Ferric
Fe^2+^: Ferrous
RBC: Red blood cell
EPO: Erythropoietin
TKI: Tyrosine-kinase inhibitor
ICI: Immune checkpoint inhibitor
IMDC: International metastatic renal cell carcinoma database consortium
OS: Overall survival
PFS: Progression-free survival
RECISTv1.1: Response evaluation criteria in solid tumors version 1.1
ECOG PS: Eastern Cooperative Oncology Group performance status
C-statistics: Uno’s Concordance Statistics
PD-L1: Programmed death ligand-1
FDA: Food and Drug Administration
CHEK2: Checkpoint kinase 2 gene
ATM: Ataxia-telangiesctasia gene
BLM: Bloom gene
MLH1: MMR gene
